# 4392 Comparative Analysis of Vascular Hemodynamics in a Young Biracial Cohort

**DOI:** 10.1017/cts.2020.116

**Published:** 2020-07-29

**Authors:** Preston Bell, Ervin Fox, Solomon Musani, Gary Mitchell

**Affiliations:** 1 University of Mississippi Medical Center

## Abstract

OBJECTIVES/GOALS: We investigated hemodynamic measures in young black and white adults below the age of 50 years to identify mechanisms that may predispose blacks to more CVE. METHODS/STUDY POPULATION: We recruited 276 young blacks and white adults in Jackson, MS (mean age: 33 ± 9 years; 70% women; 57% Black;). Participants had clinical and vascular tonometry parameters obtained. Vascular measures included carotid femoral pulse wave velocity, central and peripheral pulse pressure (CPP and PPP), characteristic impedance (Z_c_), augmentation index, and forward pressure wave. Race-specific characteristic means and proportions were tested using Student’s T- or Chi-square tests. After further sex stratification, significant variation in characteristics among sex within race were tested using nested ANOVA. RESULTS/ANTICIPATED RESULTS: Characteristics of the study group stratified by race were found to be similar. Vascular measures stratified by race revealed blacks to have significantly higher Z_c_ (p = 0.03) and PPP (p = 0.03) than whites. DISCUSSION/SIGNIFICANCE OF IMPACT: In this study of vascular hemodynamics in young black and white participants we found differences in Z_c_ and PPP. Findings suggest that differing relations between proximal aortic diameter and wall stiffness may contribute to the racial disparity in CVE in adults. This finding could offer an explanation to the beneficial effects of treatment modalities that target aortic stiffness in blacks.

